# Activities used by evidence networks to promote evidence-informed decision-making in the health sector– a rapid evidence review

**DOI:** 10.1186/s12913-024-10744-3

**Published:** 2024-02-29

**Authors:** Germán Andrés Alarcón Garavito, Thomas Moniz, Cristián Mansilla, Syka Iqbal, Rozalia Dobrogowska, Fiona Bennin, Shivangi Talwar, Ahmad Firas Khalid, Cecilia Vindrola-Padros

**Affiliations:** 1 Rapid Research Evaluation and Appraisal Lab (RREAL) – Department of Targeted Intervention, University College London, London, UK; 2 McMaster Health Forum, McMaster University, Toronto, Ontario Canada; 3 Division of Psychiatry, University College London, London, UK; 4 School of Global Health, York University, Toronto, ON Canada

**Keywords:** Evidence network, Decision-making, Health policy, Knowledge translation, Knowledge broker, Evidence dissemination, Evidence-informed

## Abstract

**Background:**

Evidence networks facilitate the exchange of information and foster international relationships among researchers and stakeholders. These networks are instrumental in enabling the integration of scientific evidence into decision-making processes. While there is a global emphasis on evidence-based decision-making at policy and organisational levels, there exists a significant gap in our understanding of the most effective activities to exchange scientific knowledge and use it in practice. The objective of this rapid review was to explore the strategies employed by evidence networks to facilitate the translation of evidence into decision-making processes. This review makes a contribution to global health policymaking by mapping the landscape of knowledge translation in this context and identifying the evidence translation activities that evidence networks have found effective.

**Methods:**

The review was guided by standardised techniques for conducting rapid evidence reviews. Document searching was based on a phased approach, commencing with a comprehensive initial search strategy and progressively refining it with each subsequent search iterations. The Preferred Reporting Items for Systematic Reviews and Meta-Analysis (PRISMA) statement was followed.

**Results:**

The review identified 143 articles, after screening 1135 articles. Out of these, 35 articles were included in the review. The studies encompassed a diverse range of countries, with the majority originating from the United States (*n* = 14), followed by Canada (*n* = 5), Sweden (*n* = 2), and various other single locations (*n* = 14). These studies presented a varied set of implementation strategies such as research-related activities, the creation of teams/task forces/partnerships, meetings/consultations, mobilising/working with communities, influencing policy, activity evaluation, training, trust-building, and regular meetings, as well as community-academic-policymaker engagement.

**Conclusions:**

Evidence networks play a crucial role in developing, sharing, and implementing high-quality research for policy. These networks face challenges like coordinating diverse stakeholders, international collaboration, language barriers, research consistency, knowledge dissemination, capacity building, evaluation, and funding. To enhance their impact, sharing network efforts with wider audiences, including local, national, and international agencies, is essential for evidence-based decision-making to shape evidence-informed policies and programmes effectively.

**Supplementary Information:**

The online version contains supplementary material available at 10.1186/s12913-024-10744-3.

## Background

Evidence networks engage researchers and stakeholders in sharing information and building multi-country relationships to enable the integration of scientific evidence in decision-making processes [1]. Evidence networks are particularly important in addressing global health challenges, where healthcare leaders and decision-makers need to make timely evidence-based decisions.

The exchange of robust knowledge linkages to policymakers, researchers, and practitioners is not a new phenomenon. In this context, knowledge translation platforms (KTP) enable interaction across various domains for knowledge production [2]. KTPs are organisational frameworks primarily focused on shaping policy decisions through the utilisation of the best available evidence and involving a strategic selection of stakeholders. KTPs are established and institutionalised to enable direct connections with authorities, public healthcare, policymakers, public agencies, non-governmental organisations and universities [3]. However, while these platforms facilitate knowledge production and dissemination, evidence networks distinguish themselves by actively engaging in connecting evidence to inform decision-making processes.

We define evidence networks as collectives of individuals dedicated to advancing evidence-informed decision-making, without the direct aim of influencing specific public policies. Evidence networks comprise networks that bring together teams locally, nationally and globally, potentially offering a way of bringing together different actors in less institutionalised and systematic ways. The use of social connections and relations facilitates evidence-use [1]. This enables researchers and decision-makers to meet to learn from one another, fostering a better understanding of decision-making processes and resource mobilisation. The literature also highlights the importance of evidence networks building capacity for sharing opportunities and exposure across traditional boundaries [1].

Despite global calls for evidence-based decision-making at policy and organisation levels, there remains a gap regarding the best approaches for scientific knowledge generation and its systematic use [3]. These gaps include limited capacity for knowledge translation platforms to evolve into permanent collaborations and a lack of dissemination in practical settings [4], alongside a discrepancy in the research that is produced, and the type of research required for decision-making [4]. In addition, there is little research specifying what kinds of evidence activities are used and a lack of clarity on how different activities can be combined and applied in different contexts [3]. The effectiveness of different network structures in diverse contexts, the role of stakeholders within these networks, and the impact of evidence networks on decision-making processes merit further attention.

There are numerous global calls to use the post-pandemic momentum to better connect and institutionalise evidence-to-policy efforts. The Global Commission on Evidence to address societal challenges launched a wake-up call to decision-makers, evidence intermediaries, and impact-oriented evidence producers to better think and structure evidence-support systems and the global evidence architecture, which included the role that multilateral organisations could have in broadening evidence-related capacities to share and use evidence [4]. These capacities would enable readiness for change by facilitating collaboration and information sharing and is in line with principles of knowledge translation. Evidence network activities expand on the foundations of knowledge translation and aim to engage communities and civil society in collaboration with researchers and decision-makers, enhancing both policy development and implementation. Networks such as the World Health Organization’s Evidence Informed Policy Networks (EVIPNet), and health sector-specific networks like Share-Net International (2023) are vital examples of transnational networks that connect people in related fields. They act as intermediaries for evidence and play a role in informing health policymaking, emphasising the importance of translating high-quality evidence into action through sharing evidence use [5]. It is imperative that evidence networks are significant in the research system to address global challenges. Without these networks, the likelihood of implementing adequate recommendations for change is limited [6].

Davies (2003) emphasised the importance of evidence networks in identifying reliable evidence sources and assessing their relevance when evaluating objectives and impact [7]. However, it is crucial to acknowledge that personal, structural, and political differences significantly impact the use of evidence-based activities [8]. To overcome these barriers, understanding activities within evidence networks such as methodological design, dissemination practices, building relationships with stakeholders and communities of practice in transparent ways, and reporting their value in organisational contexts can help create a culture shift.

A rapid evidence review design was used as the findings from the review were needed to inform decisions about the implementation of evidence networks. The rapid review design enabled the prompt synthesis of information, ensuring that the outcomes were available to inform strategic decision-making. This initiative was undertaken in collaboration with the commissioning partner, specifically the Translating Evidence into Action Thematic Working Group (E2A TWG), which operates within the larger framework of Health Systems Global (HSG). This rapid review aimed to enhance our understanding of the strategies used by evidence networks as a mechanism to translate evidence into decision-making processes. It explored how evidence networks utilised tools for analysis, assessment, evaluation, and lessons learnt.

## Methods

The design was informed by guidance for rapid evidence reviews [10]. This review followed a phased approach, beginning with a broad search strategy and subsequently expanding with each round of searches. We followed the Preferred Reporting Items for Systematic Reviews and Meta-Analysis (PRISMA) statement to guide the review design and the reporting of the methods and findings [10]. A protocol was developed before initiating the review, which served as a guide outlining the specific criteria for the study searches. This was reviewed and agreed upon by all authors, including correspondent members of the commissioning organisation. Thus, the protocol was not registered or publicly available.

### Search strategy

We identified search terms using a combination of free-text and controlled terms. We tested and refined the terms by running exploratory searches in principal databases. After a series of subsequent exploratory searches and feedback from co-authors, we developed the final search strategy. The final searches were performed in May 2023 in PubMed, the Cochrane Library, Web of Science, and Google Scholar, and included categories such as use, evidence networks, knowledge translation, health and healthcare, policy and decision-making (see Appendix 1 for the complete search strategy). Complete inclusion and exclusion criteria are described in Table 1**.**

**Table 1:** Inclusion and Exclusion Criteria

Inclusion	Exclusion
Peer-reviewed and open grey literature	PhD dissertations, books
Focus on: - Activities that evidence networks use to promote evidence-informed decision-making in the health sector - Evaluation of these activities/evaluation of the findings - Main lessons learnt in the implementation of these activities	
Studies published between 01/01/2013–01/05/2023.	Published before 01/01/2013
No geographical restrictions	
Published in English	Not published in English

### Selection criteria

The search results were imported into Rayyan, a web-based app with semi-automated features enabling the detection of duplicate publications from the different databases. The software also displays citation details, titles, and abstracts of each publication, facilitating screening [11].

The initial title and abstract screening for eligibility was conducted by GAAG, RD, TM and FB, and each record was reviewed by two reviewers independently. Following the initial screening at the title and abstract level, ST cross-checked 10% of exclusions against the inclusion criteria. Four reviewers (GAAG, RD, TM, FB) conducted full-text screening to guarantee the proper selection of the publications.

The remaining publications that met the inclusion criteria were organised and allocated randomly to the reviewers to continue full-text screening for eligibility. Four reviewers (GAAG, RD, TM and FB) independently conducted full-text screening, with 100% of included and 10% of excluded documents checked by another independent reviewer. Due to the rapid nature of the review, we only included records between January 2013 and May 2023, and the questions and search strategy were focused on identifying relevant articles that could be analysed within the review timeframe. We also excluded records that could have taken longer to review such as books or dissertations.

### Data extraction

Data extraction was conducted using an extraction form on REDCap software to organise the review process. The extraction form was first piloted and discussed with three articles from the selection, and necessary amendments were made before extracting data from the included documents. Extracted information included identifiers (e.g., first author surname, date of publication, type of article), population of interest, focus topic (e.g., maternity, child health), description of activities, assessments, results and main lessons learnt from their implementation. Data were extracted by the same four reviewers and checked by a different team member. Data extraction form is available as Appendix 2.

### Data synthesis

The researchers used framework analysis to guide the data synthesis [12]. The analysis focused on developing themes that can accurately represent the data. The categories for the framework were based on the research questions guiding the review and the information emerging from the documents. Therefore, the framework categories included types of activities, evaluation of activities and their results, as well as lessons learned from the activity implementation.

### Quality assessment

The methodological quality of the empirical articles was critically appraised using the Mixed Methods Appraisal Tool (MMAT) [13, 14]. The MMAT was developed to allow systematic reviewers to assess the methodological quality of diverse study designs, including qualitative, quantitative, and mixed methods. The assessment was performed using a scale of zero to five, considering the number of positive or negative points on five appraisal questions.

Furthermore, we used the AACODS checklist (Authority, Accuracy, Coverage, Objectivity, Date, Significance) to assess grey literature sources [15]. The score was considered based on the six sections of the checklist (Authority, Accuracy, Coverage, Objectivity, Date, and Significance). The content was assessed by three reviewers, who discussed the most appropriate questions from each section beforehand to guarantee accuracy. The score was calculated using a scale of zero to six, considering the number of positive or negative answers in each of the six sections.

The research team agreed on the importance of being transparent about the methodological quality of the articles included in the review. therefore, the team decided to avoid excluding any of the articles based on quality as these still met the predefined inclusion criteria.

## Results

### Article selection

**Fig. 1:**
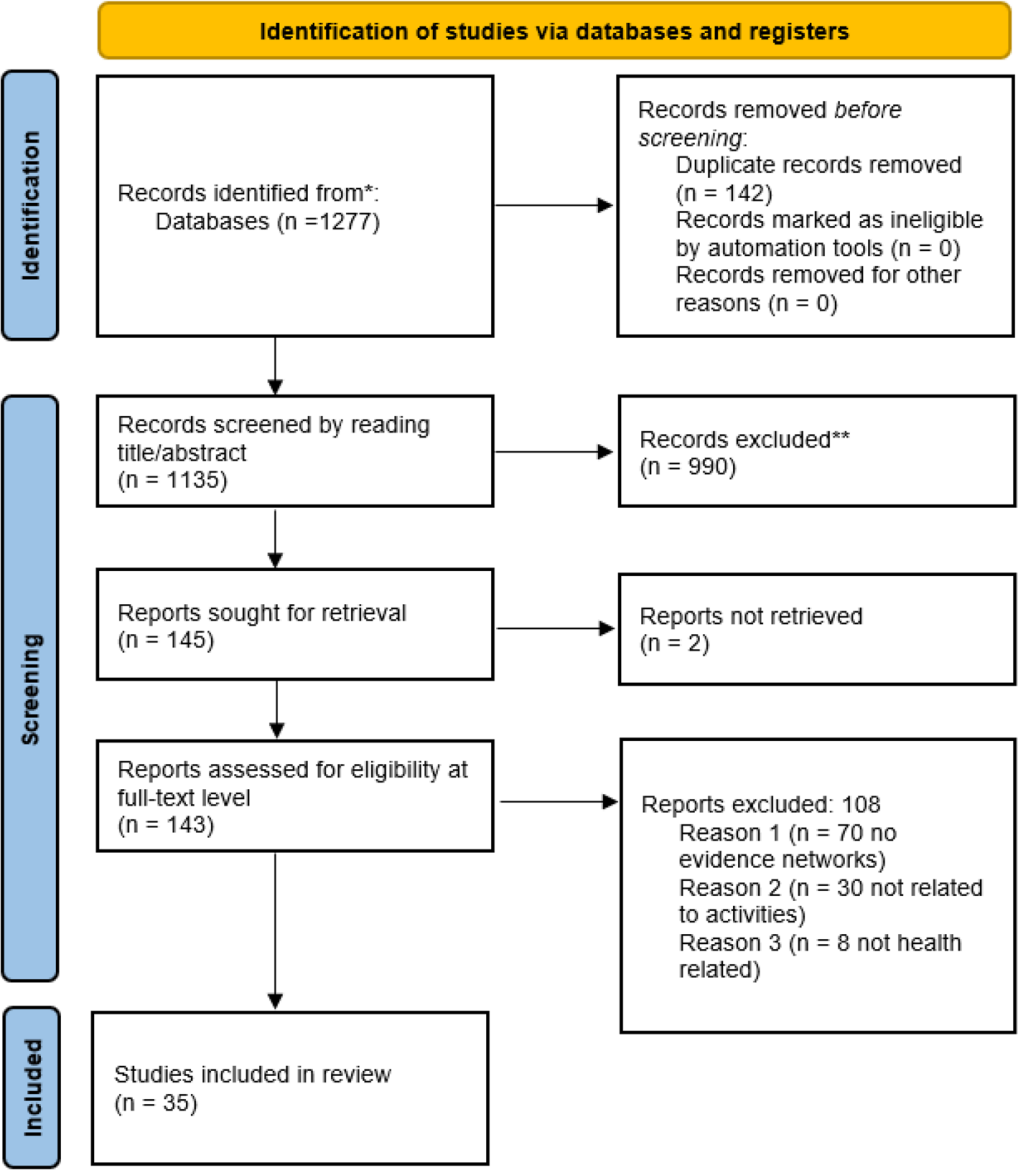
The PRISMA Flow Diagram

The initial search yielded 1277 records. After deduplication on Mendeley and Rayyan, four reviewers screened the titles and abstracts of 1135 articles. A total of 145 articles were sought for retrieval, but full texts of two articles were not available. Excluded records are available upon request. We screened full texts of 143 articles and excluded 108 because they were not about evidence networks, discussed different topics, and were not health-related. Thirty-five articles were included in the review (see Fig. 1 for the PRISMA Flow Diagram).

### Article characteristics

The 35 included articles were mainly in the United States (*n* = 14) [16–29], Canada (*n* = 5) [30–34], Sweden (*n* = 2) [35, 36], and in other single locations such as Australia, Brazil, Burundi, India, Kenya, and Nigeria, among others (*n* = 14) [37–50]. Of those included, 16 articles were qualitative studies [18, 20, 25, 28, 31–36, 44, 45, 47–50], nine were non-empirical papers [19, 22, 23, 37–39, 41–43], four were quantitative studies [26, 27, 29, 40], and six were mixed methods [16, 17, 21, 24, 30, 46]. Eleven studies reported a population of interest, which were mainly Indigenous groups [31, 34, 48], older adults [19], patients in patient group programmes [21, 27], academic faculty and researchers [40], and mental health practitioners [16], among others. The article characteristics are summarised in Appendix 3.

### Quality assessment

Overall, the quality of the included literature could be classified as high. Of the 35 included articles, 26 publications were reviewed with the MMAT [16–18, 20, 21, 24–36, 40, 44–50]. After assessing the included articles, the average score was 4.4. Four studies had a score of 4.5 [24, 27, 33, 48], presenting limitations in reporting the risk of non-response bias, interpretation of results, and how inconsistencies between quantitative and qualitative results were addressed. Overall, the quality assessment for the included empirical articles in this review was 4.9.

On the other hand, nine reports [19, 22, 23, 37–39, 41–43], which were found in the same databases, were assessed with the AACODS checklist. The main limitations identified were the lack of methodology reporting and clear coverage limits. The overall assessment score for the non-empirical articles included in this review was 5.1/6. Articles were strengthened in terms of authority by being associated with reputable organisations, presenting a reference list, and being cited by others. Furthermore, the documents were objective and relevant to the time when they were published.

### Activities

#### Types of activities

In the context of evidence networks, the activities constituted deliberate actions taken to actively enhance the use of evidence in the decision-making process. The predominant activities focussed on research-related and practical aspects, such as workshops, training sessions, and online activities, among others. Additional categories encompassed practical activities, the formation of teams, and policy influence, which are described below.

#### Research related activities

A primary method through which evidence networks facilitated evidence-informed decision-making was via research-related activities. These activities aimed to strengthen research methodology skills in participants such as policymakers and fieldworkers. Activities undertaken in the initial phases of the research process included assessing recipient needs [21], conducting early evidence assessments [22], engaging in priority audience research [21], involving stakeholders in the formulation of designing research objectives [50], conducting systematic reviews [42], creating evidence maps based on existing data, and collaborating with stakeholders in the development of research protocols [28].

In the later phases of the research process, activities included the use of research reported in papers, conference presentations and policy briefs [50]. Furthermore, there was an active collaboration with practitioners throughout the research process through interactive methods [36, 38], and the development of training seminars for evidence-based assessment and treatment, considering the needs of both patients and clinicians [16].

Additionally, several other activities were incorporated into the research process to further enrich evidence-informed decision-making. These activities included recommending survey questions, employing in-house geographic information system (GIS) mapping during a door-to-door survey [18], conducting masterclasses [48], actively disseminating research findings to relevant stakeholders and policymakers [50], advocating the dissemination, translation, and use of evidence [42] and conducting implementation and impact studies [22].

Research related to policies was conducted by engaging in policy surveillance (which was an ongoing, systematic, scientific collection and analysis of laws of public health importance) and policy ratings (a systematic, empirical method for measuring and evaluating observable policy interventions). Additionally, efforts included the development of policy briefs [50] and the initiation of collaborative research initiatives focussing on policy-related topics [44].

#### Practical activities

The practical activities most frequently mentioned included the development of training programmes [17, 19, 29, 35, 44] as the primary focus, followed by community-based and stakeholder workshops [32, 37, 44, 45, 49] and the facilitation of online webinars, portal links, discussions, and engagement on social media platforms [38, 41, 43, 48]. These activities were more common in topics such as community and rural health care [17, 35, 37], and policy research [41, 43, 45], across settings in the US [16, 17, 19, 21, 29], Brazil [45], Sub-Saharan Africa [43], Canada [32], Sweden [35], and the UK [49].

Certain activities were centred on building capacity, such as establishing network events [38], researchers developing and testing practical tools and resources designed for the development, implementation and evaluation of interventions and frameworks [19], building professional capabilities through the development and delivery of easily accessible training, resources, discussion groups, seminars and providing ongoing mentoring [16, 19, 48] alongside the development of tools and resources to contextualise and operationalise the fundamental public health function [39].

Lastly, some evidence networks provided a post-workshop or post-discussion tool or resource, including but not limited to evaluation workbooks [49], a collective book, a series of working papers, a toolkit, a blog, and engagement through e-discussions [43].

### Creation of team/task force/partnerships

Several activities were focused on the establishment of new teams, committees, and networks, including the formation of a newly established team specialising in sex, gender, and vulnerable populations (GVPs) [30]. Additionally, initiatives such as The Academic Network for Sexual and Reproductive Health and Rights Policy (ANSER) [44], a PBF Communities of Practice (CoP) [43] and Village Health Sanitation and Nutrition Committees (VHSNCs) [46]. Furthermore, a Task Force on Community Preventive Services published recommendations from an investigator-led review of community-based depression [19].

### Meetings/consultations

There were a limited number of activities which were centred around meetings or consultations. This included consultation with individual project teams, involving both formal inquiries and other informal interactions [38]. It also involved one-to-one meetings and discussions with city officials [24], as well as participation in other forums such as press for policy-level changes. Additionally, town hall meetings were held, which featured testimonies from members of the scientific and local communities [18].

### Mobilising/ working with communities

Several of the activities involved collaborating with communities. Some of these were more general activities, such as community mobilisation and coalition building [40], offering technical assistance on projects [41], conducting community listening sessions [20], designing a community participatory research project using focus groups [25], establishing robust clinic-community connections [26] and cultivating a diverse multisector partnership led by community members [25].

Additionally, there were more context-specific activities, such as identifying priorities through a “Dotmocracy” method [31], which is a decision-making technique used in group settings to identify priorities collectively. Furthermore, there were initiatives such as matching community leaders interested in health research with medical school students seeking experience in health services research [34], as well as the mobilisation of community organisations in Chinatown to form a neighbourhood children’s oral health task force [25].

### Influencing policy

Three articles centred their activities around facilitating policy maker-researcher engagement using research findings to influence policy and practice [25, 44, 50]. For instance, one study stated that the activity, which was a workshop, widened the scope of the policies they hoped to influence or execute by prompting them to explore concerns relating to the other sector [45].

Additional activities related to establishing relationships with national authorities [39] and fostering collaboration between academics and policymakers [40]. Finally, two specific activities analysed the role of stakeholder engagement and their influence on the strategic policy review process [47]. Further information can be found in Appendix 4.

### Activities evaluation

A total of 18 articles discussed the evaluation of activities used by evidence networks and their outcomes (see Table 2).

**Table 2:** Evaluation and results found in the included literature

Author	Assessment method	Results
Chinman	• The trial assessed three sets of variables: quality of performance in conducting key programming practices (e.g., goal setting, planning, evaluation), fidelity of MPC (e.g., adherence, classroom delivery, dosage), and the sexual health outcomes of participating middle school youth.	• In typical community-based settings, manuals, and training common to structured EBPs may be sufficient to yield low performance levels and moderate fidelity levels, but that more systematic implementation support is needed to achieve high levels of performance and fidelity. • In each of the 2 years, BGC sites that received MPC training plus GTO (intervention group) were found to have higher ratings of performance than sites just receiving MPC training (control group). Regarding the adherence dimension of fidelity, in year 1, sites receiving GTO were observed to have fewer instances where they did not conduct an activity of the MPC program at all compared to sites without GTO. However, both groups of sites implemented MPC activities fully only approximately half the time (55–57%). In year 2, the intervention group significantly improved their adherence, implementing MPC activities fully 92% of the time, while the control group remained similar to year 1 (55%). Overall, the second year showed more GTO impact.
Redmond	• To evaluate the effectiveness of WellConnect programs, the research team developed a secure, web-based platform that enabled WellConnect EBP leaders to enter key data elements. Each participant’s name and date of birth was used to locate their medical records from the REP.	• Of the 737 EBP workshop signups researchers were able to link medical records from the REP for 572 (77.6%) cases. There were no statistically significant differences in health outcomes between WellConnect program participants and matched controls. Falls prevention EBP participants demonstrated a 34% decreased likelihood of being admitted to the ED or hospital at 1 year of follow-up and chronic disease/pain management EBP participants demonstrated a 19% decreased likelihood compared to matched controls. This is similar to published reductions in the likelihood of ED (32%) and hospital (28%) admittance found for chronic disease management program participants.
Williams	• In-depth key-informant interviews.	• Staff from six recipients reported increased knowledge of local communities and audiences, improved efficacy, and skills to conduct media interviews, enhanced capacity to identify and train champions and influencers, and greater community partner investments. With marketing support, 90% of recipients reported increased enrolment, of which 40% exceeded self-set targets and another 40% doubled or tripled their enrolment numbers.
Goldzweig	• Process evaluation included characterization of MSFA’s involvement in each state as high (including all states selected as primary targets); low (defined as an average of less than one week (40 hours) of effort per year for the 7.42 years of evaluation or less than 297 hours); or intermediate.	• From January 2003 to May 2010, passage of primary legislation was 4.5 times as likely (95% CI 1.90, 10.68) in states with high versus low alliance involvement.
Cloke	• Conducted Focus Groups following the final workshop sessions. We explored our facilitated interactive group learning approach to enhancing the equity-sensitive evaluation of local healthcare services. To do this we asked the following: (1) What was the experience of participants of the PPP and the CIGs? (2) How was knowledge mobilisation achieved? What, why and by whom? (3) What are the key elements that enhance the process of coproducing equity-sensitive evaluations?	• Four themes were identified to illustrate how the CIG approach to delivering intensive and facilitated training supported equity-sensitive evaluations of local healthcare services that informed local decision-making by (1) creating the setting (2); establishing a common purpose (3); making connections and (4) challenging and transforming the role of evaluation.
Morais	• The facilitation team designed the evaluation and 12 months after the workshop the team reached out to participants to take part in interviews. The assessment’s goal was to elicit participants’ perceptions of the workshop’s influence on their thinking one year after the activity. Analysis of their responses provides a lens through which to view the potential contributions of CBSD as a tool for facilitating the link between knowledge production and policy-making and implementation.	• Qualitative analysis revealed five major themes in participants’ responses that indicate how the workshop experience influenced their understanding of food and transport systems and enabled their ability to incorporate this understanding into practice to support policy processes: (1) stakeholder engagement, (2) shared language and understanding of the problem, (3) interconnections, (4) dialogue across sectors, (5) use of systems thinking.
Cooke	• A mixed method evaluation of the OPEN GVP activities was conducted. Data sources included surveys and qualitative interviews of OPEN members 18 months after OPEN’s launch, an OPEN member end-of-grant survey, and GVP team meeting notes and members’ critical reflections with respect to the creation and implementation of the GVP team model.	• The 2016 end-of-project survey of OPEN teams collected data on how the OPEN teams had used GVP resources, as well as how sex, gender and vulnerable populations had been included in their research activities; six out of six teams responded. All six OPEN project teams reported using the GVP team’s recommended survey questions in their research. Four project teams had used the online sex, gender, and intersectionality learning module. Two project teams reported using individual consultations with the GVP team, however, other project teams reported that arranging formal consultations with the GVP team was not necessary as they had a team member who was part of the GVP team and able to provide the necessary support. None of the OPEN teams mentioned using the modified HEIA tool in their research.
Springs	• The project evaluation included an online survey and debriefing sessions after each phase of the process and as indicated by the CRPs. The survey measured indicators of engagement in research as described in the Patient-Centered Outcomes Research Institute (PCORI) Engagement Strategy Rubric (i.e., reciprocal relationships, partnership, co-learning, transparency, honesty, and trust).	• The survey revealed that the approach to evidence synthesis measured high on all aspects of engagement. Participation also improved CRPs confidence in engaging with the health care system, developed greater empathy, and understanding of others in the community and increased interest in using science or research in advocacy efforts. The researchers measured the tangible skills developed as part of the training, with most participants indicating that they felt confident in their abilities to develop a research question, search the medical literature, read a journal article, and identify a population, intervention, or outcome in a journal article.
Maar	• Participants were invited to contribute through semi-structured telephone interviews using open-ended questions to discuss their perspectives and experiences about the program. Three different, but complementary, interview guides were developed, interviewing 1) Community contact, 2) Medical students, and 3) Faculty Supervisors.	• The research showed that the CETR program had the potential to maintain positive trust-based relationships between medical learners and Indigenous communities. Furthermore, medical students experienced the importance of relationship building in Indigenous research. Yet, to ensure the sustainability of the CETR program several strategies are required including, (1) Formalize the supervisory relationship between faculty and student, (2) Initiate ethics application early, (3) Identify sustainable funding, (4) Track and evaluate research output.
Stajic	• At the end of each Masterclass, participants were encouraged to complete a short paper-based evaluation form. • A formal evaluation to answer the research question ‘*Can a short educational intervention strengthen research capacity in the Aboriginal Community-Controlled Organisation sector*?’ was undertaken to better understand the reach and impact of these Masterclasses.	• This evaluation found that the foundation-level Masterclass, ‘Understanding Research’, was requested most often by the ACCHO sector, providing an indication of the sector’s current research literacy needs and that the priority of health services is service delivery and not undertaking or leading research. Most survey and interview participants had established careers in Indigenous health, suggesting that their developed research capacity is likely to be sustained in the sector. Participants described a developed understanding and confidence in research, leading to increased willingness to participate in research and, importantly, a greater sense of empowerment in interactions with external research partners.
Pullyblank	• RE-AIM framework was helpful in evaluating public health interventions, including dissemination and scaling of projects in a real-world setting. “Reach” was assessed using survey data as well as the REDCap database. Patient characteristics were used descriptively to assess Reach. “Effectiveness” was evaluated through completion status. “Adoption” was assessed from internal documents to summarize engagement of partner organizations and peer leaders. We evaluated “Implementation” using internal documents to assess fidelity, as well as capturing the Plan-Do-Study-Act (PDSA) changes that occurred throughout the implementation process. “Maintenance” included sustainable strategies we implemented which were gleaned from reviewing internal documents.	• REACH: 474 individuals enrolled in a DSMP workshop (34 workshops offered), and 306 individuals enrolled in CDSMP workshop (29 workshops offered). • EFFECTIVENESS: Completion percentages among those enrolled in the DSMP or CDSMP were 74.7 and 79.4%, respectively. • ADOPTION: A total of 617 referrals to the program had been made by clinicians within the health care network between 2017 and 2019, with 15% of enrolees recruited through the EHR in 2019. • IMPLEMENTATION: By the end of 2019, participants could learn about the program through traditional media, social media, bulk communication sent through the patient portal, their provider, a call from Living Well, or from partner organizations. • MAINTENANCE: Living Well continuously sought funding to sustain the program until a payment model could be implemented either by the health care system or third-party payers.
Sharma	• Cross-sectional quantitative survey of 140 village health sanitation and nutrition committees (VHSNCs) designed to assess six parameters of VHSNCs, including their formation, composition, meeting frequency, activities, supervisory mechanisms, and funds’ receipt and expenditures. • Researchers developed a semi-structured questionnaire in the local language consisting of questions related to each of the six parameters, questions on supervisory mechanisms (visits by officials, e.g., medical officers and program managers, and visit frequency), funds’ receipt and expenditures. Additionally, we asked about the role of accredited social health activists (ASHA) in the VHSNC meetings, and the issues and challenges faced.	• The number of members in VHSNCs in most of the studies, including our study, were in accordance with the guidelines, except Odisha. The VHSNC guidelines recommended the presence of representatives from the marginalized classes in the committee. However, most studies echoed that marginalized classes are frequently poorly represented or not taken seriously. This poor representation of marginalized classes in the meetings connotes the caste and power dynamics in villages. Unlike most of the studies, we reported that VHSNC meetings occurred regularly. In our study, most of the VHSNCs had a fixed date for holding the meetings which may help prompt the members to attend it regularly. Our study echoed the previous findings that funds were limited, and there was a delay in payments to VSHNCs. We found inadequate supportive supervision and monitoring visits by the government officials, (e.g., medical officers, child development project officer).
Buchwald	• One-on-one exit interviews with the scholars and mentors, as well as open-ended questions on surveys administered to scholars and representatives from community organization partners about their overall experience and satisfaction with the program. The quantitative evaluation was based on the results of the Clinical Research Appraisal Inventory (CRAI), augmented by a comprehensive module on PCOR and CER skills and competencies developed by PCORP faculty.	• Of the 22 scholars who completed the post-evaluation, 23% completed their projects before training ended, 64% were still working on it, and 9% did not plan to complete their projects. Some barriers identified to completing the project included institutional issues, EHR data extraction issues, patient attrition, Institutional Review Board issues, shifts in organizational priorities, change of roles, and lack of a home institution mentor due to turnover or role transitions. Overall, 32% of scholars reported that PCORP improved their skills significantly and 59% reported moderate improvement. PCORP’s overall usefulness to the scholars was rated as very good by 36%, good by 46%, and fair by 18%. Satisfaction with the overall experience in PCORP was rated as very satisfied by 32%, satisfied by 59%, and dissatisfied by 9%.
Bertone	• Analyses of online discussion forums.	• Performance Based Financing (PBF) is expanding rapidly in sub-Saharan Africa; while the PBF Community of Practice’s own contribution is difficult to ascertain, it has established itself as the main platform for knowledge exchange and development on PBF. Some early analyses of the online forum discussions have confirmed the focus on a specific policy domain, the collective sharing of a technical repertoire and the emergence of an identity and community spirit, all key features of a de facto CoP.
Driedger	• Participants were asked a series of questions to address perceptions around important aspects that modellers and public health practitioners faced during the 2009 H1N1 outbreaks regarding mathematical modelling and pandemic responses.	• Three main challenges to developing model-informed public health decisions emerged through the following themes: (1) models need to be relevant to public health priorities (2); clear communication and plain language about what models can (and cannot) do is needed; and (3) the importance of developing strong working relationships through collaboration and integration.
Guinaudie	ACCESS OM was assessing the innovative ways in which SDM strategies might foster effective integrated knowledge translation (IKT) in a youth mental health research setting. A working group with representatives from these two councils and each participating site was created to provide feedback on key outcome domains and measures that should form part of the ACCESS OM’s quantitative assessment protocol.	• The inclusion of representatives highlighted important criteria for choice of assessments tools (e.g., short tools with youth-friendly language, domains that go beyond symptoms), and item response options: the demographic questionnaire has an expanded set of options for sexual orientation and gender identity. Informal feedback from site service providers indicated that young service users across sites appreciated the range of options provided for these two questions and seen this as an indicator of ACCESS OM sites being “safe spaces”. A notable SDM activity involved youth and family/carers in working groups and consultations to influence the redesign of research consent forms, which had been reported as being too lengthy and difficult to understand.
Malcolm	• Post seminar face-to-face interviews were conducted individually with several participants who were asked to talk about the experience of completing the EBAT seminar. For each EBP, clinicians were asked about their knowledge in that particular evidence-based practice, their perceived skill in implementing that practice, and how often they use that practice when it is clinically appropriate.	• Looking across EBP, results suggest that clinicians consistently grew in their knowledge, skill, and ability to implement EBP when clinically appropriate.
Smith	• Publicly available policy materials were studied in both case studies, with a focus on contributions to the European Commission consultation on smokefree policies and the English consultation for the Marmot Review. Team attempted to investigate relationships among network members (‘network’ referring to organisations that provided a customised policy response). Semi-structured narrative interviews with politicians, researchers, advocacy groups, and other individuals involved in policy discussions related to each case study were used to collect qualitative data.	• The analysis suggested that the ways in which actors organised themselves to employ (and deploy) evidence in policy debates was crucial to understanding its impact. • Case Study 1 illustrated how researchers, health professionals, advocates and policymakers could actively collaborate in public health policy development, with public health advocates drawing on scientific evidence to strengthen their arguments; researchers working with advocates to better understand the strategic policy context and provide the kinds of research required to advance policy goals; and policymakers seeking contact with researchers and advocates to develop policies which were backed by available evidence. • Case Study 2, in contrast, demonstrated the difficulties of employing evidence in policy contexts for which advocates, and advocacy coalitions were lacking. In the context of a lack of leadership and an unfavourable political climate, no organised network emerged and levels of trust between those involved in trying to effect policy change was low.

### Main lessons learnt in the implementation of these activities

The articles identified a diverse variety of lessons and valuable insights learned from the implementation of activities aimed at promoting evidence-based decision-making. A description of the main lessons can be found below.

### Interdisciplinary research

The articles emphasised the significance of interdisciplinary research in incorporating a more comprehensive spectrum of viewpoints and expertise, ultimately facilitating policy transformation [30, 32, 36, 37, 45, 47]. Previous research found that interdisciplinary teams were particularly useful for large and complex research projects that involved several different sub-projects and priorities were addressed, necessitating distinct areas of expertise [30].

Establishing an interdisciplinary and intersectoral Community of Practice (CoP) could offer a potential solution for bridging the gap between researchers, policy-makers, and healthcare professionals [32]. An interdisciplinary CoP of this nature could help to guide collaborative efforts between researchers and public health officials. Within such a network, the models could be developed rapidly and flexibly as policy questions were formulated and modified. To form such a network, it was necessary to identify individuals possessing a diverse range of skills to ensure effective collaboration [32].

### Training

Five authors highlighted the importance of education and training in the successful implementation of activities [16, 17, 29, 48, 49]. For example, in the case of Indigenous ACCHO staff capacity, training played a pivotal role in not only enabling their active participation and collaboration in research, but also in empowering them to take an active role in identifying research questions and priorities, conducting research and evaluation activities, and translating findings into practice [48]. Although online learning may be useful, the most important sources contributing to the development of research skills were recognised as individual project-based skill application, in-person learning, and peer networking [17].

In one particular article, it was found that delivering training equipped participants with the tools and confidence to address their organisation’s aims and objectives of reducing health inequalities. This was achieved by mobilising knowledge from various stakeholders to coproduce evaluations for their local services [49]. In another article, it was reported that clinicians found seminars to help foster a positive attitude towards evidence-based practice. Additionally, the article highlighted the significance of ongoing training for experienced clinicians as a means to increase the likelihood of delivering the highest quality care [16]. Lastly, one article noted that deficient performance within village health committees was associated with inadequate training [29].

### Trust building and regular meetings

To ensure mutual alignment and understanding of shared goals, the importance of conducting regular meetings was underscored as a means to promote transparency and cultivate a deeper level of inter-network trust [29, 33, 44, 47]. To ensure the successful implementation of activities, alignment with the objectives and priorities of diverse stakeholders was imperative [17]. Additionally, informal meetings were highlighted as a way to offer researchers an opportunity to network with stakeholders such as policymakers, such meetings improved the potential for fostering partnerships and increased the likelihood of research findings being used by various stakeholders [44].

Another article found that the majority of challenges associated with the implementation of activities stemmed from the need to adapt to and collaborate with other organisations, each with its distinct agendas and demands [36]. As such, maximising avenues for communication and collaboration remained essential to achieving alignment between different actors, however, it was essential to acknowledge that, at times, there might not be a good fit between these actors and their respective priorities. Certainly, the articles underscored that, to achieve successful implementation, activities must be aligned with the organisational goals and priorities of different stakeholders [17, 33].

### Media engagement

Two articles emphasised the importance of involving the media in disseminating research findings and enhancing the probability of policy adoption [41, 44]. Researchers should actively engage with the media to ensure widespread public dissemination of key research findings and to underscore key issues [44]. Additionally, another article similarly stressed the potential of social media as a valuable tool to disseminate knowledge and information related to evidence-based healthcare, including new research findings and critical appraisal of current practices [41].

### Community - academic – policymaker engagement

Selected articles cited the importance of fostering engagement between the community, researchers, healthcare professionals and policymakers. Researchers should identify ‘knowledge gaps’ for policy-making and target their research to address these gaps effectively [44]. This can help to ensure that health policies being developed are firmly based on evidence and effective in tackling the most relevant problems and the most vulnerable populations.

It was also highlighted that engaging policy and decision-makers in the early stages of the research, particularly during the identification of priority-setting processes and throughout data collection, strengthened the connection between evidence and policy implementation. The authors also proposed that involving policymakers from the beginning helps to increase their willingness to use research findings, even in cases where these findings contradicted their expectations or current policies, ultimately enhancing the credibility of the research findings [50].

Five articles underscored the importance of establishing well-defined research questions and carefully informed objectives as essential components of conducting evidence-based decision-making [30, 33, 47, 48, 50]. In four articles, it was highlighted that community-academic research brokers play a crucial role in fostering mutual alignment and ensuring that research aligns with the needs of the community and exerts influence on policymakers as well [18, 23, 28, 46]. Others highlighted the importance of clarifying outcomes that are of importance to patients and communities, especially those that are underrepresented in the literature. They also stressed the importance of identifying comparisons between interventions that resonate with these patients and communities [28].

Some articles also noted that the implementation of activities highlighted the importance of collaboration between different stakeholders in co-producing programmes [20, 32, 38, 44]. The significance of involving affected communities should not be underestimated to ensure that the community’s needs and expectations are incorporated into the activities implemented [18, 28, 29, 46].

Furthermore, five articles highlighted the importance of providing expert supervision during the design and implementation of community programmes to ensure fidelity to the programme model [24, 29, 34, 43, 46]. To that end, it is necessary to establish robust connections based on transparent communication among communities, clinicians, researchers, and policymakers [22, 23, 26, 34, 38, 48].

## Discussion

The aim of this review was to synthesise the existing evidence on the activities that evidence networks used to promote evidence-informed decision-making, while also drawing lessons learned from their implementation and evaluation. The review identified numerous activities relating to research, practical training, teams or partnership formation, community mobilisation and working with communities and policy influence.

### Research activities

Research activities serve as a primary mechanism by which evidence networks facilitate evidence-informed decision-making, and these activities can occur at different stages of the research process. To increase program uptake, especially in underrepresented groups, practitioners disseminating evidence-based interventions may consider implementing a marketing support system based on recipient needs and research focused on priority audiences [21]. This distinctive approach is centred on not only identifying needs and implementing informed methods to address them but also on simultaneously building capacity [21]. Conducting early evidence assessments is recognised as a reliable and adaptable method for assessing the foundation of the best available evidence related to an intervention. This process can inform short-term decision-making and serve as a guiding framework for further research in the longer term [22, 51]. The use of early evidence assessments identified policy interventions with a strong evidence base, which facilitated knowledge translation efforts and later on policy adoption [22].

However, early evidence assessments require close surveillance as the evidence can evolve rapidly. This means that subsequent assessments need to be prepared in a relevant way that captures the scope effectively [22]. This activity indicated that the policy research continuum was best approached in a multi-phased and systematic way, the benefits of adopting this approach resulted in improved decision-making, enhanced research quality, and more effective policy development.

The collaborative involvement of practitioners can play a central role in the promotion of evidence-informed decision-making [36]. The relevance of applied research tends to be greater when knowledge has been co-produced with stakeholders, including practitioners, and researchers. The inclusion of practitioners has the potential of accelerating the adoption of evidence-based recommendations, and enhancing relevance since evidence might align with the practical needs of practitioners, making the research more relevant and impactful [36].

Furthermore, researchers can foster engagement with practitioners by employing interactive approaches at different stages, ranging from mapping the research problem to implementation processes and ultimately disseminating results. As such, practitioners can serve as “informants, recipients, endorsers, commissioners or co-researchers” [36]. This highlights the practical benefits of collaboration, such as improved policy outcomes or enhanced effectiveness in addressing real-world problems. However, co-production such as this researcher-practitioner collaboration can be difficult due to the different agendas and demands stemming from the practitioners’ and researchers’ respective contexts [36, 38].

Research-to-policy linkages have been described as generally weak and characterised by a lack of communication and engagement among researchers, communities, and policymakers [50]. Meaningful engagement was seen as a crucial step in translating research evidence into policy and practice. The active engagement of stakeholders and policymakers during the initial stages of the research helped shape the research design and aims [50]. Therefore, establishing direct interactions with policymakers who will ultimately rely on the forthcoming evidence can be crucial for achieving success. Policy retreats and workshops that enable direct face-to-face engagement between researchers and decision-makers are considered more effective than sharing conference proceedings or providing information about the research results in which they were not actively engaged in [50].

It is important to acknowledge however that direct engagement may not always be feasible, often requiring a well-established, long-term relationship between evidence networks and policy makers. Consequently, researchers frequently find themselves relying on the dissemination of results, with the hope of eliciting a reaction or provoking a response. Nonetheless, it was notable that research organisations and evidence networks are frequently undervalued by policymakers, either because they are perceived as lacking an understanding of the policy-making process or due to challenges in effectively communicating research evidence [50].

Systematic reviews are an important tool for promoting evidence-informed decision-making, as they can obtain and appraise evidence in an objective, reliable and transparent manner. This method was cited as being particularly significant when they are tailored to the specific context, as evident in the African continent. This region is characterised historically by a limited research capacity, high disease burden and fragile health systems, therefore systematic reviews emerge as indispensable tools [42]. Evidence-based decision-making is enhanced by facilitating the translation of evidence into various languages, thereby broadening the reach of policy audience. However, its crucial to acknowledge that systematic reviews prioritise certain types of research, such as quantitative scientific evidence [42].

Evidence mapping, like systematic reviews, follows a structured and replicable approach, making it particularly valuable for uncovering hidden links or patterns between interventions and different populations [21]. This method favours descriptive qualitative data and its tabular categorisation, offering policymakers a broad overview of evidence, although it may not capture the precise, detail of a statistical meta-analysis.

The involvement of community research partners (CRPS) and stakeholders enhances the value of such activities. CRPs empower and enable non-researchers from the community to participate in and coproduce the research. Meanwhile, stakeholders inform the protocol’s development and explain findings that hold importance to the community [28]. The research was further strengthened by translating the results of the evidence synthesis into an online interactive tool, this ensures that the collaborative results are accessible and meaningful to community partners.

Educational courses focused on research serve as an important means of promoting evidence-informed decision-making among stakeholders, as participants are more likely to adopt research findings [16, 48]. An example of this impact was illustrated by the Masterclass Program offered to strengthen the research capacity of staff within Aboriginal Community-Controlled Health Organisations (ACCHOs) [48]. Participants in the study described gaining critical thinking skills, an increased understanding of research and the use of evidence, an increased willingness to participate in research, and greater confidence in their research abilities [48]. Fundamentally, equipping staff with research knowledge is crucial for them to effectively advocate for and facilitate community-driven research, promote culturally sensitive practices, and ensure accountability to local communities [48]. Nevertheless, such activities are heavily dependent on the availability of funding, which is often limited in state-funded services, and time constraints that are common due to typically demanding workloads in the health sector [16, 48].

### Practical activities

Evidence networks also engage in practical activities to advance evidence-based decision-making. Often these activities will take the form of training programmes or community-based workshops [17, 19, 29, 32, 35, 37, 44, 45, 49] or they involve offering online webinars, engaging in discussions and utilising social media platforms [38, 41, 43, 48]. The Academic Network for Sexual and Reproductive Health and Rights Policy (ANSER) was developed to address the gap between research and policy in SRHR [44]. It is a global platform for SRHR policy research, education and healthcare delivery. The ANSER network initiates collaborative research on SRHR policy-related topics, by developing a portfolio of education and training programmes, and fostering interaction between SRHR researchers and policymakers [44]. In an evaluation of community-based system dynamics (CBSD) workshops, authors found that the participatory modelling approach, which aimed to build stakeholders’ capacity to collaboratively address complex challenges, effectively engaged individuals from various academic and professional backgrounds. Furthermore, it successfully fostered trust among the involved participants [45]. Although this method enables a holistic exchange of perspectives, it should be acknowledged that the method is not without resistance and can lead to disagreements and conflict between participants [52]. Nevertheless, creating space for disagreement also fosters constructive dialogue, identifying intersections, shared perspectives, and the development of a deeper shared language [45].

Another form of practical activities that can significantly contribute to advancing evidence-based policy involves enhancing professional capacity. This is achieved through development and delivery of accessible training, resources, discussion groups, seminars and providing ongoing mentoring support [16, 19, 48]. In a training programme on Evidence Based Practice (EBP) for effective child and adolescent mental health practice, it was observed that conducting the training on-site and incorporating it into regularly scheduled meetings significantly enhanced its adoption ( [16]. Clinicians in community mental health clinics were constrained due to time pressures and potentially penalised for prioritising training over their clinical duties. Therefore, it is vital to make training as easily accessible as feasible. Furthermore, uptake was incentivised by providing food at training sessions to ensure participants would not have to choose between taking breaks and attending training [16]. Nevertheless, the findings suggest that participants found it challenging to commit to 90-minute sessions, and occasionally had to miss them due to crisis appointments [16]. One possible solution could involve facilitating online participation or selectively inviting clinicians to relevant modules. It was emphasised that online learning was an especially useful resource. However, the skill application acquired through experiential and in-person learning, and peer networking are identified as the most important factors in developing of research skills [17].

### Network

An important aspect of the activities involved collaboration by forming networks, teams and partnerships to facilitate the production and dissemination of knowledge [23, 30, 38, 42–44, 46]. In 2017, South African researchers came together to form Cochrane Africa, an endeavour to aimed at coordinating effort to build capacity for conducting systematic reviews and promote the use of best evidence. They also aimed to translate evidence into other languages (especially French and Portuguese) to inform healthcare decision making [42]. Cochrane Africa focuses on five activities: 1) developing context-relevant systematic reviews based on research gaps, consultation and needs; 2) capacity-building research skills; 3) advocating the dissemination translation and use of evidence; 4) building partnerships to promote locally led evidence-informed healthcare and 5) facilitating evidence-based decision-making, enhancing evidence availability, fostering research networks and communities, and aiding the translation of evidence into different languages [42]. However, numerous challenges hinder effective collaboration across countries within the network and languages, including language barriers, variances in communication channels, cultural differences, the lack of financial support and low motivation levels [42, 53]. Another significant issue revolves around researchers’ limited understanding of the policy process and how to engage policymakers, coupled with policymakers’ lack of experience in understanding how evidence is generated [52]. Evidence networks operate distinctively from KTPs in facilitating and sharing evidence-informed decision-making and offering the potential for a greater readiness for change [2] . KTP platforms are widely recognised for more broadly transferring research into policy. This review shows that evidence networks could contribute to the development of KTPS, as seen in initiatives such as EVIPnet. Furthermore, collaborating with stakeholders and expanding to include evidence networks can be viewed as an opportunity to better link and consolidate research to action, thus significantly contributing to the evolving discourse in this field.

### Community mobilisation

Community engagement and mobilisation are vital for incorporating the community’s perspectives and needs into policy deliberation and generating community advocacy for policy change [25]. Additionally, it was noted that taking policy action at the intersection of research, business and community interests can be a useful method for overcoming popular opposition to evidence-based policy change [23]. To achieve consensus, community networks can engage deliberative practices during gatherings, operationalising methods such as ‘dotmocracy’ [31]. Dotmocracy is a consensus-based process of voting with stickers to identify priorities in smaller groups before reconvening in a single larger group to present their priorities. These smaller groups compile and discuss their priorities all together until a consensus is reached across the groups, ultimately leading to a consensus-based conclusion for the round table discussion [31]. This was an effective decision-making process which was grounded in conflict resolution, considering diverse perspectives to drive transformative change. Nevertheless, it must be underpinned by respect, mutual recognition of rights and a sense of cooperation and collaboration.

### Engagement

This paper identified different types of engagement within evidence networks that improved the use of evidence, such as social media, webinars and workshops to disseminate information to broad audiences. However, we found challenges regarding meaningful engagement, such as inconsistencies in evidence tools, keeping them up to date, and measuring impact. In addition, collaboration between stakeholders, policymakers and researchers was reported as an important barrier to engagement in evidence-based decision-making, particularly using evidence-based activities in meaningful ways [54]. Often, decision-makers are those with the most power and influence, therefore, to increase engagement and render the process more equitable it is important to actively involve others [25].

One study [54] recommended the use of ‘champions’ or ‘knowledge brokers’ to give weight to evidence activities, which can also help reduce inequalities and empower community stakeholders. However, the study found that decision-makers do not perceive this as solely their responsibility, emphasising the importance of providing evidence [55]. This may have implications for how evidence activities are used. There is a need to understand the ways in which relationships between stakeholders, policymakers and communities are enhanced as well as look at evidence activities. Methods such as network analysis which include communities and local knowledge are useful to mitigate these and strengthen evidence networks [54].

### Strengths and limitations

The review was strengthened by having four reviewers screening articles and cross-checking exclusions, and by using two different quality appraisals such as the MMAT and the AACODS to assess the quality of the included literature.

While the use of a rapid evidence review design proves valuable in time-sensitive contexts where evidence is required promptly to inform decision-making, it is important to note that this review may not be as exhaustive as a systematic review. Thus, the review was restricted by resource and time limitations, meaning that only a limited number of databases and websites were accessed within a restricted timeframe. Specific subject headings, keyword terms and synonyms may have been missed.

Hence, it is recommended that future studies address the methodological gaps identified in this review and current research. This could involve expanding the number of selected databases and assessed records. Additionally, we recommend that future reviews are guided by principles of stakeholder involvement and co-production [18] to include relevant stakeholders, such as experienced knowledge brokers, academics, policymakers, and evidence network participants. Their involvement could provide valuable input on any potentially overlooked literature, explore diverse insights to effectively meet objective, as well as aid in the analysis and validation of findings.

Finally, it was notable that the evaluation of these activities to promote evidence-informed decision-making was not extensively detailed in the available literature. In total, 18 of the included papers reported an assessment of their activities, but, for many, the reported results were limited to a description of the appraisal method and lacked additional details.

## Conclusion

Evidence networks are of paramount importance to assist the development, dissemination and uptake of relevant, high-quality research evidence activities and its implementation into policy and programmes. This interdisciplinary approach is particularly vital in tackling complex global challenges and leveraging the current momentum in research to drive progress. Evidence networks serve as a crucial initiative in connecting individuals and organisations with similar objectives. However, these networks are not without their challenges, as highlighted in this review. The challenges encompass the coordination of efforts among diverse stakeholders, navigating working across countries internationally and language barriers, ensuring consistency of research, effective knowledge dissemination to relevant stakeholders, building research and community capacities, feasible evaluation of activities, and sustainable funding. To further advance the impact of evidence networks, the next step is to share the efforts of evidence networks and activities undertaken to wider audiences such as local, national and international agencies who are committed to knowledge exchange and evidence-based decision making. By addressing these challenges and embracing opportunities for growth, evidence networks can continue to be instrumental in shaping evidence-informed policies and programmes.

### Supplementary Information

**Supplementary material 1.**

**Supplementary material 2.**

**Supplementary material 3.**

**Supplementary material 4.**

**Supplementary material 5.**

## Abbreviations

AACODS: Authority, Accuracy, Coverage, Objectivity, Date, Significance
ANSER: The Academic Network for Sexual and Reproductive Health and Rights Policy (ANSER)
CBSD: Community-Cased System Dynamics
CoP: Communities of Practice
EVIPNet: Evidence Informed Policy Networks
MMAT: Mixed Methods Appraisal Tool
PRISMA: The Preferred Reporting Items for Systematic Reviews and Meta-Analysis
REDCap: Research Electronic Data Capture
SRHR: Sexual and reproductive health and rights
VHSNCs: Village Health Sanitation and Nutrition Committees
