# Supplementary material for: Activities used by evidence networks to promote evidence-informed decision-making in the health sector– a rapid evidence review
Source: BMC Health Serv Res. 2024 Feb 29;24:261. doi: 10.1186/s12913-024-10744-3 (PMC10903073; doi:10.1186/s12913-024-10744-3)
Supplement: Supplementary file 1 — Supplementary material 1. [file 12913_2024_10744_MOESM1_ESM.docx]

***Appendix 1.*** **Search strategy**

**Themes**

*#1 Evidence network*

Evidence network OR knowledge translation OR translational medical research OR evidence-based OR evidence brief OR capacity building OR deliberative dialogue OR evidence community OR evidence cluster AND healthcare OR health

#2 *Health*

healthcare OR health

*#3 Decision-making*

Decision Making OR Policy formulation OR policy making OR prototyping OR policy OR public policy OR health policy OR health planning OR program evaluation OR health system plans OR policy application OR policy translation OR policy adoption

**Strategy**

For the initial exploration of results to determine sensitivity versus breadth we are looking at:

1 + 2 + 3

**Evidence network OR knowledge translation OR translational medical research OR evidence-based OR evidence brief OR capacity building OR deliberative dialogue OR evidence community OR evidence cluster AND healthcare OR health AND healthcare OR health AND Decision Making OR Policy formulation OR policy making OR prototyping OR policy OR public policy OR health policy OR health planning OR program evaluation OR health system plans OR policy application OR policy translation OR policy adoption**

**Date range:** (01-05-2013 / 01-05-2023).

**Language:** Only English.

**Location:** No restrictions**.**

**Website searching**

Medline (PubMed): 157 results (TI only)

WOS: 244 results (TI only)

The Cochrane library: 776 reviews (TI or AB)

Google Scholar (top 100 hits): 80,900 results.
