# Supplementary material for: Activities used by evidence networks to promote evidence-informed decision-making in the health sector– a rapid evidence review
Source: BMC Health Serv Res. 2024 Feb 29;24:261. doi: 10.1186/s12913-024-10744-3 (PMC10903073; doi:10.1186/s12913-024-10744-3)
Supplement: Supplementary file 2 — Supplementary material 2. [file 12913_2024_10744_MOESM2_ESM.pdf]

Activities used by evidence networks to promote evidence-informed

Please complete the survey below.

Thank you!

Surname / first author	________________
Date of publication (Year)	________________
Location (Just country name) / For specified places within a country, use the next question.	________________
Please specify place if necessary.	________________
Type of article	◯ Qualitative study<br>◯ Quantitative study<br>◯ Mixed methods<br>◯ Non-empirical report (i.e. review, commentary)
If empirical study, type of study design	________________
Is there a population of interest?	◯ Yes<br>◯ No
If so, which one?	________________
What is the focus topic? (e.g., maternity, child health etc.)	________________
What activities are used by the evidence network to promote evidence-informed decision-making?	________________
Were the activities evaluated?	◯ Yes<br>◯ No
Please describe the assessment.	________________
Please summarise the main findings.	________________
What are the main lessons learnt in the implementation of these activities?	________________

Other comments
