# Supplementary material for: Activities used by evidence networks to promote evidence-informed decision-making in the health sector– a rapid evidence review
Source: BMC Health Serv Res. 2024 Feb 29;24:261. doi: 10.1186/s12913-024-10744-3 (PMC10903073; doi:10.1186/s12913-024-10744-3)
Supplement: Supplementary file 3 — Supplementary material 3. [file 12913_2024_10744_MOESM3_ESM.docx]

***Appendix 3.*** **Main article characteristics**

| **Surname / first author** | **Year of publication** | **Study location** | **Type of article** | **Population** | **MMAT/AACODS score** |
| --- | --- | --- | --- | --- | --- |
| Amy Keir (28) | 2018 | Unspecified | Non-empirical report | Not specified | 4.75/6 |
| Belza (20) | 2014 | USA | Non-empirical report | Older adults | 4.75/6 |
| Bertone (27) | 2013 | Burundi & Sub-Saharan Africa | Non-empirical report | Not specified | 6/6 |
| Cacari-Stone (16) | 2014 | USA | Qualitative study | Not specified | 5/5 |
| Chinman (19) | 2016 | USA | Quantitative study | Adolescents | 5/5 |
| Cloke (23) | 2023 | United Kingdom | Qualitative study | Not specified | 5/5 |
| Cooke (30) | 2017 | Canada | Mixed methods | Not specified | 5/5 |
| Crowshoe (37) | 2021 | Canada | Qualitative study | Indigenous people groups | 5/5 |
| Cusworth Walker (34) | 2022 | USA | Qualitative study | Not specified | 5/5 |
| Dedra S. Buchwald (21) | 2023 | USA | Mixed methods | Not specified | 5/5 |
| Driedger (26) | 2014 | Canada | Qualitative study | Not specified | 5/5 |
| Erika B. Fulmer (9) | 2020 | USA | Non-empirical report | Not specified | 5/6 |
| Goldzweig (32) | 2013 | USA | Non-empirical report | Not specified | 5/6 |
| Guinaudie (41) | 2020 | Canada | Qualitative study | Young people at mental health services | 4.5/5 |
| Hewitt (14) | 2018 | Wales | Non-empirical report | Not specified | 4.75/6 |
| Jakab (29) | 2021 | Unspecified | Non-empirical report | Not specified | 4.75/6 |
| Jessani (39) | 2016 | Kenya | Quantitative study | Academic faculty | 5/5 |
| Kerry Darr Gabbert (33) | 2022 | USA | Mixed methods | Not specified | 4.5/5 |
| Kevin Grumbach (35) | 2017 | USA | Qualitative study | Not specified | 5/5 |
| Kristin Pullyblank (36) | 2022 | USA | Quantitative study | Not specified | 5/5 |
| M. E. Nyström (13) | 2018 | Sweden | Qualitative study | Not specified | 5/5 |
| Maar (38) | 2018 | Canada | Qualitative study | Indigenous people groups | 5/5 |
| Malcolm (15) | 2019 | USA | Mixed methods | Mental Health Clinicians | 5/5 |
| Mbuagbaw (11) | 2018 | Africa | Non-empirical report | Not specified | 4.75/6 |
| Morais (24) | 2021 | Brazil | Qualitative study | Health researchers | 5/5 |
| Ogbe (18) | 2018 | Various | Qualitative study | Not specified | 5/5 |
| Redmond (42) | 2020 | USA | Quantitative study | Patients | 4.5/5 |
| Schneider (25) | 2022 | Various | Non-empirical report | Not specified | 6/6 |
| Sharma (31) | 2022 | India | Mixed methods | Not specified | 5/5 |
| Smith (40) | 2018 | EU and UK | Qualitative study | Not specified | 5/5 |
| Springs (12) | 2019 | USA | Qualitative study | Not specified | 5/5 |
| Stajic (17) | 2019 | Australia | Qualitative study | Indigenous people groups | 4.5/5 |
| Urban Markström (22) | 2014 | Sweden | Qualitative study | Not specified | 5/5 |
| Uzochukwu (10) | 2016 | Nigeria | Qualitative study | Not specified | 5/5 |
| Williams (8) | 2022 | USA | Mixed methods | Patients | 5/5 |
