# Supplementary material for: Activities used by evidence networks to promote evidence-informed decision-making in the health sector– a rapid evidence review
Source: BMC Health Serv Res. 2024 Feb 29;24:261. doi: 10.1186/s12913-024-10744-3 (PMC10903073; doi:10.1186/s12913-024-10744-3)
Supplement: Supplementary file 4 — Supplementary material 4. [file 12913_2024_10744_MOESM4_ESM.docx]

**Appendix 4. Activity categories**

PRACTICAL ACTIVITIES

- Training.
- Implementation of support tools.
- On-site Technical assistance.
- Developing strategies & training.
- HAN researchers developing and testing practical tools and resources for the development, implementation and evaluation of interventions and frameworks (e.g., RE-AIM) for their dissemination and sustainability.
- Building professional capacity by developing and delivering accessible, state-of-the-science trainings and resources.
- Workshops; Community-based system dynamics workshops; Stakeholders' workshops; Organisation of workshop.
- Compiling an evaluation workbook and providing it to all participants of the workshops as a resource.
- Developing an online module using a web-based learning management system.
- Production of policy briefs and distribution to policy makers and programme managers.
- Stakeholders dividing themselves into smaller groups of 8-10 to discuss three facilitation questions.
- Being provided with guides, portal links to resources & ongoing mentoring.
- Workshops with a network of 23 public health researchers and implementers from seven institutions across six countries (Zambia, South Africa, Sweden, Tanzania, Uganda and Guatemala) who have been collaborating and meeting regularly on the theme of CHSs. The key themes of action were grouped into an initial set of lenses, presented at the start of the workshop, and through various forms of representation (such as rich pictures and text) these lenses were defined and elaborated, including the research methodologies typical to each.
- Developing a portfolio of education and training programmes on SRHR policy (also policy activity).
- Designing PCORP, a stakeholder-driven educational and experiential training program designed to train scientists, clinicians, and other health care professionals involved in quality improvement, research, and evaluation initiatives (could also be a research activity).
- Launching an online discussion group.
- Different knowledge activities being organised by the CoPKM: workshops, a collective book, a working paper series, a toolkit, a blog, e-discussions, etc.
- Social media use, like the FOAM movement.
- Swedish training program being launched in order to introduce Assertive Community Treatment (ACT) in all parts of the country based on Evidence Based Practice.
- Webinars are an innovative approach that enable 'live' contact with network schools at a national level.
- Network events: The value of face-to-face contact with schools was recognised at the network's launch event, and subsequently an annual event for schools has been held.
- 90-minute interactive EBAT seminars conducted across 15 weeks.
- Developing tools and resources to contextualise and operationalise the essential public health functions (EPHFs) integral to health systems strengthening and health security efforts.

CREATION OF TEAM/ TASK FORCE/ PARTNERSHIPS

- Creating a new sex, gender and vulnerable populations (GVPs) team.
- Building partnerships to promote African-led evidence-informed healthcare and creating opportunities for the network to grow.
- Developing The Academic Network for Sexual and Reproductive Health and Rights Policy (ANSER).
- Constituting the Village Health Sanitation and Nutrition Committees (VHSNCs).
- Launching PBF Communities of Practice (CoP).
- Pursuing shared priorities through coordination, collaboration and knowledge sharing to build more resilient public health systems.

MEETINGS/ CONSULTATIONS

- Consulting with individual OPEN project teams, both through formal inquiries and other more informal interactions.
- One-on-one meetings with city officials, and other forums to press for policy-level changes; One on- one discussion of results and advocacy with policy makers and programme managers
- Town hall meetings featuring both science and community testimonies

RESEARCH ACTIVITIES

- Conducting recipient needs assessment
- Conducting priority audience research
- Task Force on Community Preventive Services publishing recommendations from a HAN investigator-led review of community-based depression.
- Constructing recommended survey questions to be used by OPEN project teams collecting their own survey data
- In-house geographic information system (GIS) mapping in a door-to-door survey
- Involving stakeholders in designing objectives of a research and throughout the research period
- Active dissemination of research findings to relevant stakeholders and policymakers:
  - research reports
  - peer reviewed papers
  - conference presentations; Conference presentations of findings
  - policy briefs
- Evidence mapping of arts-based healthcare interventions to better understand the quantity and quality of existing evidence.
- Priming stakeholders with an initial presentation at gatherings.
- Including both stakeholders and CRPs participating in the development and implementation of evidence synthesis protocols.
- Masterclass Program comprising three Masterclasses - Understanding Research, Undertaking Research and Research Evaluation delivered free of charge.
- Producing context-relevant systematic reviews identified through a consultative process with end-users based on their needs, priorities and acknowledged research gaps.
- Building capacity to conduct and use systematic reviews.
- Advocating for the dissemination, translation and use of evidence.
- Early evidence assessments.
- Policy surveillance (ongoing, systematic, scientific collection and analysis of laws of public health significance).
- Implementation studies.
- Policy ratings (systematic, empirical method to measure and evaluate observable policy interventions)
- Impact studies.
- Initiating collaborative research on SRHR policy-related topics.
- 1) describing health services research,  evidence-based medicine, and the historical and regulatory evolution of PCOR and CER; 2) articulate methods, findings, and implications of PCOR and CER; 3) critically appraise PCOR and CER  studies; 4) identify relevant data sources and their strengths and  weaknesses; 5) apply results of PCOR and CER to clinical domains;  6) describe quality metrics and their use in health care and payment models; and 7) describe diverse stakeholders and best practices for engaging patients and stakeholders in all phases of PCOR  and CER
- Collaborative involvement of practitioners in the research process using interactive forms during the following stages: (1) mapping of the research problem and  its manifestation in practice, and formulation of research  questions; (2) planning, creation of interventions and  choices of design; (3) investigation, test, follow-up and implementation processes; (4) analyses, reflection and learning; and (5) reporting on and dissemination of results,  including further implementation of studied intervention.
- Use of free open-access medical education (FOAM).
- Bespoke Student Health and Well-being Reports reporting each member school's student survey data by gender and year group, with national data for comparison.
- Network research being translated for schools through research briefs.
- Developing evidence-based assessment and treatment training seminar considering the needs of patients and clinicians.
- Linking EBP participant records with health outcomes documented in medical records.

MOBILISING/ WORKING WITH COMMUNITIES

- Mobilising communities and coalitions.
- Identifying priorities for Indigenous PHC action through the "Dotmocracy" method.
- Matching Indigenous distributed education sites where community leaders have expressed interest in exploring health research questions with NOSM (med school) learners who seek experience in health services research (also research activity).
- Holding 15 community listening sessions.
- Conducting a community participatory research project that used focus groups.
- Developing a diverse multisector partnership spearheaded by community members.
- Providing a quick-response team.
- Mobilising community organisations in Chinatown to form a neighbourhood children's oral health task force.

INFLUENCING POLICY

- Changing laws and public policy.
- Policymakers and donors seeking evidence from researchers (could also be a research activity)
- Facilitating policy maker-researcher engagement in best ways of using research findings to influence policy and practice.
- Briefing San Francisco policymakers on the science demonstrating adverse health outcomes of SSB consumption and the evidence of effectiveness of regulatory and pricing policies in reducing consumption.
- Fostering interaction between SRHR researchers and policy-makers
- Investing and collaborating with national authorities (Ministries of Health) to establish and strengthen NPHIs and promote the EPHFs.
- Mapping the individual 'academic faculty-policymaker' connections that underlie the institutional networks and identifying individual persons playing a hub role and therefore serving as academic KBs.
- Analysing stakeholder engagement in context of EU Council recommendation on smoke-free environments.
- Analysing efforts to influence a UK Government Strategic review of Health inequalities.

OTHER

- Influencing the individual at the point of decision.
- Modifying environmental factors to modify behaviours.
- Working towards the elimination of underlying causes.
